# Mitigating local over-fitting during single particle reconstruction with SIDESPLITTER

**DOI:** 10.1016/j.jsb.2020.107545

**Published:** 2020-08-01

**Authors:** Kailash Ramlaul, Colin M. Palmer, Takanori Nakane, Christopher H.S. Aylett

**Affiliations:** a Section for Structural and Synthetic Biology, Department of Infectious Disease, Faculty of Medicine, Imperial College Road, South Kensington, London SW7 2BB, United Kingdom; b Scientific Computing Department, Science and Technology Facilities Council, Research Complex at Harwell, Didcot OX11 0FA, United Kingdom; c Medical Research Council Laboratory of Molecular Biology, Cambridge CB2 0QH, United Kingdom

**Keywords:** 2D/3D, 2/3-dimensional, Cryo-EM, electron cryo-microscopy, EM, electron microscopy, FSC, Fourier shell correlation, LAFTER, Local Agreement Filter for Transmission EM Reconstructions, SNR, signal to noise ratio, Cryo-EM, Local resolution, Noise suppression, Real-space filter, Over-fitting

## Abstract

Single particle analysis has become a key structural biology technique. Experimental images are extremely noisy, and during iterative refinement it is possible to stably incorporate noise into the reconstruction. Such “over-fitting” can lead to misinterpretation of the structure and flawed biological results. Several strategies are routinely used to prevent over-fitting, the most common being independent refinement of two ***sides*** of a ***split*** dataset. In this study, we show that over-fitting remains an issue within regions of low local signal-to-noise, despite independent refinement of half datasets. We propose a modification of the refinement process through the application of a local signal-to-noise filter: SIDESPLITTER. We show that our approach can reduce over-fitting for both idealised and experimental data while maintaining independence between the two sides of a split refinement. SIDESPLITTER refinement leads to improved density, and can also lead to improvement of the final resolution in extreme cases where datasets are prone to severe over-fitting, such as small membrane proteins.

## Highlights

• Over-fitting occurs in regions of low local signal-to-noise despite independent half-set refinement.
• We show that real space filtering according to SNR during refinement prevents overfitting.
• We describe an algorithm, SIDESPLITTER, that achieves this while maintaining independence between half-sets.
• SIDESPLITTER can also improve the final resolution in cases of severe over-fitting.

## Graphical abstract

**Figure:**
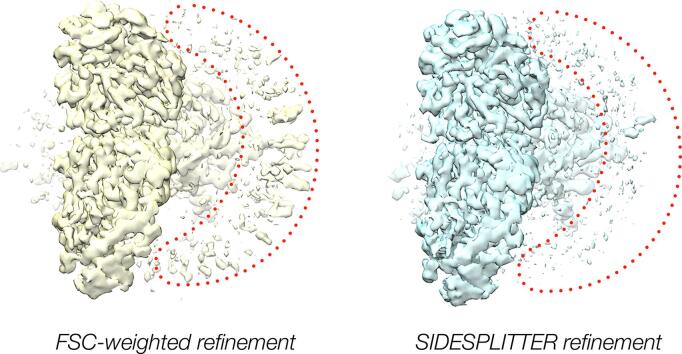

## 1 Introduction

### 1.1 Improved versions of the iterative projection matching approach underlie current single particle 3D reconstruction

Technological developments are enabling cryogenic electron microscopy (cryo-EM) reconstruction of macromolecules to increasingly high resolutions (Frank, 2017, Elmlund et al., 2017), providing a viable alternative to crystallography for larger (>100 kDa) complexes. These structures are determined by single-particle analysis, which entails the three-dimensional (3D) reconstruction of the molecule’s electron scattering density from thousands or millions of individual projection images of randomly oriented particles (Elmlund and Elmlund, 2015, Carazo et al., 2015, Vilas et al., 2018b, Lyumkis, 2019).

Reconstruction of the macromolecular structure of interest is usually carried out in reciprocal space and relies on the Fourier projection theorem. The theorem states that the Fourier transform of an object’s projection is equivalent to a slice through the centre of the Fourier transform of the projected object in 3D (Bracewell, 1956). The correct alignment of each particle image is essential for the reconstruction, and therefore the accurate estimation of the angular and positional parameters represents the defining problem of single-particle analysis.

Most current computational procedures used to achieve alignment are derived from improvements to the projection matching process (Penczek et al., 1994). Experimental images are compared to *in silico* projections of a 3D reference map at multiple known angles and assigned orientation parameters based on their similarity. Direct assignment, maximum likelihood, Bayesian *maximum a posteriori*, and several other less well-defined statistical approaches, have been applied to better estimate similarity and reduce bias within this process (Scheres, 2012a, Carazo et al., 2015). Iterated reconstruction and angular assignment allows the optimisation of the parameters assigned to each projection, leading to a stable and representative 3D reconstruction if the sample is sufficiently homogenous and the initial 3D reference sufficiently accurate for convergence.

### 1.2 The independent 3D refinement of two halves of a split dataset is typically used to avoid over-fitting through global filtering based on their agreement

Cryo-EM data are exceptionally noisy. The principal cause of this is the necessity to limit the electron dose used to acquire each image because of radiation damage. This results in “shot noise”: stochastic sampling of the electron scattering probability distribution. Conformational and compositional variation between particles (including that due to radiation damage), results in heterogeneity and is also a substantial source of noise. Finally, errors in parameter estimation (e.g. in contrast transfer function determination), optical aberrations (e.g. coma), and temporal variations (e.g. uncorrected beam-induced motion), also contribute noise. The overall result is an extremely low signal-to-noise ratio (SNR) (Liao and Frank, 2010, Penczek, 2010, Vilas et al., 2018b).

During iterative independent refinement both the noise and signal from the data will be incorporated into each successive pair of structures. It is essential to suppress noise before the next alignment, otherwise images will be aligned to features due to the noise as well as those due to the signal. Successive iterations would then incorporate noise stably into the reconstruction. This phenomenon is termed over-fitting (Grigorieff, 2000, Scheres and Chen, 2012), and can lead to misinterpretation of the reconstruction (Scheres, 2012, Chen et al., 2013). Because the SNR decreases with increasing resolution, this has typically been handled by applying a low-pass filter in Fourier space to down-weight the noisy higher frequencies.

**Fig. 1:**
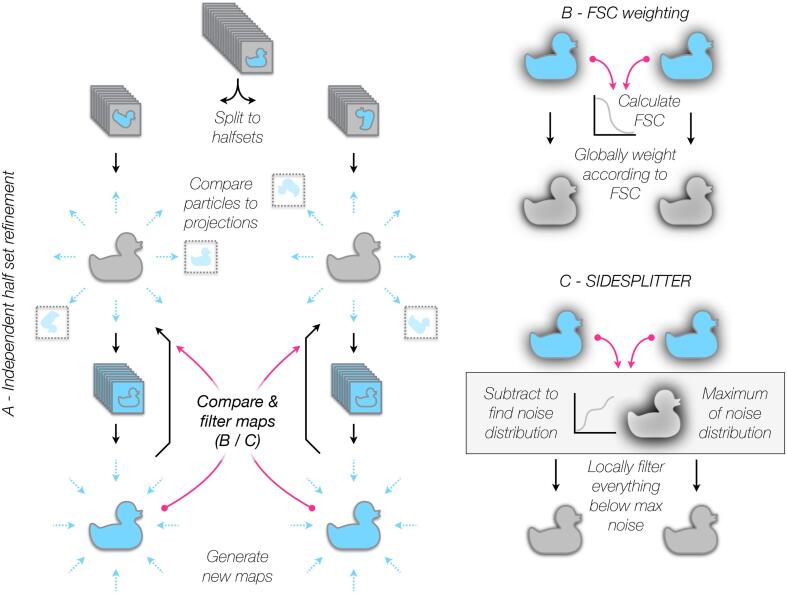
The application of SIDESPLITTER during the refinement process. Flow diagrams illustrating (A) pseudo-independent half-set projection matching refinement, (B) the application of FSC weighting as currently common in refinement, and (C) the application of SIDESPLITTER during such a refinement. Sources of information transfer between the pseudo-independent sides of the refinement are indicated with magenta arrows.

The widely-accepted measure of agreement during EM reconstruction is the Fourier Shell Correlation (FSC) between two halves of a single dataset (Harauz and Van Heel, 1986, Rosenthal and Henderson, 2003, Scheres and Chen, 2012). Particle images are randomly split into two half datasets. Each half is then refined separately using identical procedures, as one side of an independent pair of reconstructions (Fig. 1A) (Grigorieff, 2000, Scheres and Chen, 2012, Henderson et al., 2012). The cross-correlation between Fourier components in successive resolution shells of each half-map is then calculated (Fig. 1B). At low resolution, the correlation between half-maps is expected to be high (approaching unity); at high resolution, the correlation should oscillate around zero.

The exact parameters for global filtering before alignment remain somewhat controversial (Van Heel and Schatz, 2005). Ideally, the measure of agreement needed for cryo-EM reconstructions is that between the experimental reconstruction, comprising both signal and noise, and the true structure, comprising only signal. Although a noiseless structure cannot be obtained, a theoretical estimate of this correlation can be calculated from the FSC, which is denoted C_ref_. This is argued to represent the cryo-EM equivalent of the crystallographic figure of merit, and therefore a C_ref_ value of 0.5 (which occurs when the FSC value is 0.143) represents a resolution criterion consistent with crystallography (Rosenthal and Henderson, 2003).

Because the sides of the refinement are kept independent, the FSC calculation between the two sides should remain statistically unbiased; low-pass filtering at the threshold resolution at each iteration will therefore prevent noise incorporation at frequencies beyond the resolution cut-off.

### 1.3 Globally filtered reconstruction methods must be expected to over-fit local noise, as they cannot take account of local variations in SNR

The current practice of low-pass filtering based on a certain resolution cut-off and weighting according to the FSC is only capable of excluding noise at a higher resolution than the chosen cut-off from being incorporated into the refinement. The phenomenon of over-fitting has therefore become intimately linked to the evaluation of the resolution of cryo-EM reconstructions, where the resolution represents the maximum spatial frequency at which the information in the map is considered reliably interpretable (Penczek, 2010).

Whereas in crystallography, crystal packing of ordered units results in a relatively constant degree of order, and therefore resolution, throughout the unit cell, in cryo-EM reconstructions there is much greater variability. This arises from several causes: heterogeneity in the particles used for reconstruction, non-uniform 3D reconstitution of Fourier components in Fourier space (Grigorieff, 2000), and inaccurate estimation of particle orientations. The result is observed in real space as locally variable SNR within the reconstruction, and referred to as “local resolution” (Cardone et al., 2013). This effect is particularly pronounced in regions of a structure which display conformational flexibility or partial occupancy.

It has been shown previously that over-fitting occurs preferentially in regions of lower SNR (Stewart and Grigorieff, 2004). Several methods for evaluation and treatment of local resolution in cryo-EM reconstruction have previously been proposed (Stewart and Grigorieff, 2004, Cardone et al., 2013, Chen et al., 2013, Kucukelbir et al., 2014, Vilas et al., 2018a, Ramírez-Aportela et al., 2020), however none have provided a way to minimise over-fitting during the reconstruction process.

### 1.4 Local SNR filtering can mitigate local over-fitting during refinement

The challenge in 3D reconstruction is to make the best use of the available signal without incorporating noise. Therefore, we should aim to maximise the contribution of the available signal at all spatial frequencies during refinement, without over-fitting in regions of lower SNR (and hence lower local resolution). This requires a local filtering approach. Recently, we introduced a new de-noising algorithm, LAFTER, which reduces the contribution of noise to cryo-EM reconstructions using two sequential real-space filtering steps (Ramlaul et al., 2019). LAFTER appeared to us to be particularly promising as a filter to prevent over-fitting, since it reduces noise by an optimal amount as judged by comparison to C_ref_, the most common standard used during independent half-set refinement (Rosenthal and Henderson, 2003). LAFTER was not applicable to the over-fitting problem during 3D refinement, however, as it shares information between the two half-set reconstructions, violating the requirement for the two halves to remain independent.

In this paper, we present SIDESPLITTER, a heavily modified adaptation of the LAFTER SNR filter optimised to process both ***sides*** of a ***split*** refinement, which we successfully integrate into 3D refinement. SIDESPLITTER maintains independence between the two sides of the refinement, sharing only the statistical properties of the noise distribution (Fig. 1C; Supp. Fig. S1). We show that over-fitting is more pronounced in regions of lower local SNR, using both experimental reconstructions and synthetic datasets with explicitly-defined local resolution. We further show that the application of the SIDESPLITTER noise-minimisation algorithm during iterative 3D refinement minimises over-fitting in poorly-resolved regions whilst retaining signal, and can improve the attainable resolution for structures with severe over-fitting.

## 2 Methods

### 2.1 Justification and aims

Our first key aim is to minimise the residual noise during the refinement process, which biases the alignment on both sides of a split refinement and thereby results in over-fitting. We aim in particular to reduce residual noise within regions of lower local SNR that are not currently protected by the global filtering approaches in widespread use. Any noise within these regions is capable of biasing the alignment in successive iterations, and should therefore be suppressed. This represents an evolutionary improvement on current binary “masking” procedures to suppress noise outside of a chosen region of a structure. Such masking approaches cannot account for differences in local SNR, and therefore either simply incorporate noise or waste useful signal. We aim to incorporate as much useful signal as possible, while suppressing as much problematic noise as possible. Our second key aim is to maintain the independence between the two sides of the split refinement, since violation of this independence would lead to overestimation of the resolution of the reconstruction and would risk global over-fitting.

The first aim requires a local filter that is capable of substantially suppressing noise while retaining signal. The second requires that we avoid the use of a shared local window or a shared resolution map for both half-sets, as these readily generate artefactual correlations between the sides of a split refinement (Supp. Fig. 1). Only global information can be shared without generating spurious correlations, and therefore a filter must either estimate SNR from the map alone (difficult in masked refinements as no regions of pure noise are available), or must use only global statistics to establish the similarities and differences between the two sides for this purpose.

To achieve these two aims we have adapted our previous SNR filter based on local agreement (LAFTER) (Ramlaul et al., 2019). To do this we have modified it to share only global statistics on the noise distribution in each shell, and no local statistics, between the independent sides. Therefore SIDESPLITTER will not result in any greater leakage of information between sides of the refinement than FSC weighting (Supp. Fig. 1) (Grigorieff, 2000, Scheres and Chen, 2012, Henderson et al., 2012).

### 2.2 Necessary assumptions

We assume that the noise is statistically independent between the two half-sets of the refinement process, while any agreement between the sides represents signal. This assumption is necessary as the properties of the noise distribution are estimated from the difference between sides. We note that this same assumption underlies current global filtering approaches during refinement.

Secondly, the signal is assumed to vary smoothly in real space. This is necessary as we operate upon isolated frequency bands from the reconstructed maps in Fourier space, and any discontinuities in the signal would affect all frequency bands. This assumption is justifiable as macromolecular structures are known to be smooth at all resolutions accessible to cryo-EM.

Finally, as we use the maxima of the overall noise distribution between the half-sets to define the extent of the noise within the refinement, the noise must be well-distributed over the density. Regions of exceptionally strong noise would be expected to result in over-aggressive filtering, although this will not necessarily be detrimental to the refinement process. We note that aggressive filtering is emphatically a lesser evil than the alternative.

### 2.3 The SIDESPLITTER algorithm

SIDESPLITTER was derived from the LAFTER SNR-based local filter. Extensive modifications have been made to the original algorithm in order to maintain as much independence between the two sides of the refinement as possible. In order to make SIDESPLITTER compatible with modern refinement algorithms (Scheres, 2012b, Punjani et al., 2017), the amplitude spectrum is normalised to that of the input volume but tapered according to C_ref_. The overall approach follows a similar, two-pass, pattern to LAFTER, first normalising resolution shells in order to allow the SNR to be evaluated independently of resolution, and then truncating the frequencies contributing to each voxel at the resolution at which the signal falls below the maximum of the noise.

Initially, each input half map is masked and Fourier transformed, and C_ref_ in each Fourier shell at resolution r is calculated using the FSC as follows (Rosenthal and Henderson, 2003):

$$\mathrm{FSC}\left(r\right)=\frac{\sum_{i}F_{1,i}F_{2,i}^{*}}{\sqrt{\sum_{i}\left|F_{1,i}\right|\sum_{i}\left|F_{2,i}\right|}}$$

$$C_{\mathrm{ref}}\left(r\right)=\sqrt{\frac{2FSC(r)}{1+FSC(r)}}$$

where $F_{1,i}$ represents the value of a Fourier coefficient of the first half map at a point i within the shell. The amplitude spectrum of each half map is calculated as the average amplitude $\left|F\right|$ of the n Fourier coefficients in each shell:

$$S_{\mathrm{in}}\left(r\right)=\langle \left|F\left(r\right)\right|\rangle =\frac{1}{n}\sum_{i}\sqrt{F\left(i\right)F^{*}\left(i\right)}$$

The spectrum for each half is stored, to be reapplied at the end of the process so that the grey-scale can be maintained in the output half maps.

SIDESPLITTER then normalises the (unmasked) half-maps. First, resolution shells are isolated from the two half maps by band-pass filtering. The half maps are transformed into Fourier space, and for each resolution shell, the Fourier coefficients are weighted using an eighth-order Butterworth band-pass filter (Butterworth, 1930):

$$F_{\mathrm{out}}\left(r\right)=F_{\mathrm{in}}\left(r\right)\left(\sqrt{\frac{1}{1+{\left(\frac{r}{h}\right)}^{16}}}-\sqrt{\frac{1}{1+{\left(\frac{r}{l}\right)}^{16}}}\right)$$

$F_{\mathrm{in}}\left(r\right)$ and $F_{\mathrm{out}}\left(r\right)$ represent the complex Fourier coefficients at radius r in the original transform and the band-passed output respectively, while h and l represent the high and low cut-off frequencies. For each resolution shell, the two half volumes are then transformed to real space after the band-pass filter has been applied. The power of the combined map at this resolution, T, and the power of the noise, N, are calculated from the sums and differences of the voxel values respectively:

$$T=\sum_{\mathrm{xyz}}{\left(v_{1,xyz}+v_{2,xyz}\right)}^{2}$$

$$N=\sum_{\mathrm{xyz}}{\left(v_{1,xyz}-v_{2,xyz}\right)}^{2}$$

$v_{1,xyz}$ and $v_{2,xyz}$ represent the magnitude of the voxels from the two half-volumes at position xyz, and the sum is over all voxel positions within the mask (as provided by the user, or a simple spherical mask otherwise).

The proportional contributions of the noise and the signal to the total power are calculated as follows:

$$P_{N}=\frac{N}{T}$$

$$P_{S}=1-P_{N}$$

The voxel values in real space are normalised (to make them comparable for the second filter) by the resolution shell width and the root mean squared value of the total power at that resolution:

$$v_{\mathrm{out},xyz}=v_{\mathrm{in},xyz}\frac{\left(h-l\right)}{\sqrt{T/n}}$$

Incorporation of further high-resolution shells is terminated either when the FSC within the mask falls below 0.143 or $P_{S}$ falls below 0.05 (whichever comes first). After all resolution shells have been processed, the series of band-passed, noise-weighted maps for each half volume is summed in real space, combining the isolated resolutions to yield a pair of normalised half volumes, which should remain statistically independent over resolution, having only been scaled by simple multiplication in each shell.

In the filtering step the noise-suppressed half volumes from the first filter are transformed into the Fourier domain, and then each is low-pass filtered at every resolution that was considered in the previous step. Low-pass filtering is performed similarly to the band-pass filtering described above, using an eighth-order Butterworth response (Butterworth, 1930):

$$F_{\mathrm{out}}\left(r\right)=F_{\mathrm{in}}\left(r\right)\sqrt{\frac{1}{1+{\left(\frac{r}{h}\right)}^{16}}}$$

Each pair of low-pass-filtered half-maps is transformed back into real space. The observed maximum noise between half volumes is found as the greatest difference between corresponding voxels in the half volumes, for all voxel coordinates xyz within the masked region:

$${\mathrm{noise}}_{\max}=\max_{\mathrm{xyz}}\left(\left|\frac{v_{1,xyz}-v_{2,xyz}}{2}\right|\right)$$

An expected upper bound on the maximum of the noise distribution, assuming the noise is normally distributed, is also calculated, according to:

$$E\left[{\mathrm{noise}}_{\max}\right]\leq RMSD\left(\frac{v_{1,xyz}-v_{2,xyz}}{2}\right)\sqrt{2}\sqrt{\log n}$$

where $RMSD\left(\frac{v_{1,xyz}-v_{2,xyz}}{2}\right)$ represents the root-mean-square deviation of the halved voxel differences within the mask, and n is the number of voxels considered. The formula is reproduced from a reference text by Dr. van Handel (van Handel, 2014), and represents a corollary of Jensen’s inequality (Jensen, 1906).

Whichever noise bound is greater is used; the expected bound is a better estimate where the noise is close to being normally distributed, whereas the observed bound acts as a fall-back for cases in which the noise is strongly non-normally distributed, which is common when symmetry averaging operations have been applied. The noise values are halved in each case to account for the fact that they will be compared to voxel values in each half map separately.

Starting at the highest resolution considered, each voxel in each half volume is tested. If its value is greater than the noise bound at the current resolution, then that value is assigned to the corresponding voxel in that output half volume. If its value is lower than the noise maximum, the corresponding voxel in the output half volume is left un-assigned, and re-considered at the next (lower) resolution. Voxels that have already been assigned at higher resolution are excluded from consideration at lower resolutions, so that each voxel in each output half map is assigned to its value at the highest resolution at which its signal is greater than the maximum noise.

In order to preserve the grey-scale from the input, the previous normalisation operation is reversed. The output half maps are band-pass filtered at each resolution, and the real-space values are multiplied by the root mean squared value of the total power at that resolution ($\sqrt{T/n}$) and divided by the resolution shell width (h-l). The band-passed maps are then summed together.

Finally, the maps are masked and the amplitude spectrum $S_{\mathrm{out}}\left(r\right)$ is calculated as before. The Fourier coefficients in each shell are multiplied by the ratio of the input and output spectra to restore the original grey-scale, and multiplied by C_ref_:

$$F\left(i\right)=F\left(i\right)C_{\mathrm{ref}}\frac{S_{\mathrm{in}}\left(r\right)}{S_{\mathrm{out}}\left(r\right)}$$

The output maps are then transformed back to real space and passed into the 3D refinement program as reference volumes for it to use in its next iteration.

### 2.4 Generation of synthetic data for testing

For the synthetic test macromolecular structure, density for the AAA+ ATPase p97 was generated from an atomic model (PDB ID 1R7R; Huyton et al., 2003), which was used to create a benchmark synthetic dataset. Density from the molecular model was generated using phenix.fmodel from the PHENIX suite of programs (Adams et al., 2010) followed by the CCP4 suite program fft (Collaborative Computational Project, 1994). Each protomer of the model map was then masked individually and explicitly low-pass filtered to a given resolution stepwise around the ring (0.0125, 0.025, 0.05, 0.1, 0.2 and 0.35 cycles per voxel), creating a defined local resolution gradient around the ring. The SNR with resolution was explicitly maintained throughout using Gaussian noise. These locally filtered volumes were then modulated with a tau-factor falloff taken from the experimental SWR1-nucleosome dataset (Willhoft et al., 2018). The RELION utility relion_project was used to generate projections from the synthetic volumes, where the orientational distribution, CTF and noise parameters were taken from the experimental SWR1-nucleosome dataset (Willhoft et al., 2018) following a methodology similar to that previously reported for analysis of γ-secretase (Bai et al., 2015). The final projection images therefore represent a noisy, CTF-convoluted experimental dataset with a non-uniform distribution of projections exactly equivalent to the donor dataset.

### 2.5 Experimental datasets used for testing

Experimental datasets corresponding to EMD-9849 / EMPIAR-10264 (Lee et al., 2019), EMD-20806 / EMPIAR-10330 (Kim et al., 2019) and EMD-4038 (Wilkinson et al., 2016) were kindly made available by Y Lee, J, Kim, and DB Wigley, to test the applicability of the SIDESPLITTER refinement process within experimental refinement workflows. In the case of EMPIAR-10330, the deposited STAR file lacked the amplitude contrast column, which was therefore set to 0.1, and micrographs were regrouped into 84 groups of roughly 100 particles each, according to defocus.

We believe that further validation of our method will best be facilitated by widespread use, and would actively encourage users to communicate any results from particularly difficult or interesting SIDESPLITTER refinements, especially heterogenous, conformationally flexible, poorly alignable, or contaminated datasets.

### 2.6 3D reconstruction pipeline during SIDESPLITTER testing

Reconstructions were performed using RELION 3.0 (synthetic data) and an alpha version of RELION 3.1 (experimental data). All data were treated to an “auto-refinement” of half-sets in RELION, starting from known angular positions, but at low resolution, and otherwise with default parameters apart from the “--solvent_correct_fsc” flag, which was applied throughout, and a mask generated in RELION, which was applied with the “--solvent_mask” flag. We used the RELION parameter “--zero_mask 0”, which fills masked regions with noise according to the calculated noise spectrum rather than zeros. This is more computationally intensive but proved to yield a particularly notable improvement over the (less computationally intensive) alternative with SIDESPLITTER (Supplementary Fig. 3).

In the case of synthetic data, for which the angles needed examination on each cycle to maintain the correct subunit positions, RELION 3.0 was run for single iterations at a time, each called with the “--continue” flag. Between each iteration, SIDESPLITTER was applied to the unfiltered half maps output by the refinement job, and the “_data.star” files processed with a python script that ensured that the particles had not moved to an adjacent subunit in the ring.

For experimental data, through the use of an alpha version of RELION 3.1, we were able to make use of an additional new feature built into the relion_refine program. When called with an additional argument (--external_reconstruct), relion_refine calls an external program to perform reconstruction of the half maps after each iteration of 3D refinement. We used this as a hook to allow us to filter the half maps after they have been reconstructed and before the next iteration begins. RELION then reads back the filtered half maps, and optionally the updated FSC curve, for use in the next iteration.

### 2.7 Experimental model refinement

For model fitting in the LAT1 example, we began with the original authors’ deposited model (Lee et al., 2019; PDB ID: 6JMQ). Our RELION reconstruction was post-processed as normal (using FSC-weighting), and the model was rigidly fitted into the volume using the “Fit in map” function in UCSF Chimera (Pettersen et al., 2004). The atomic coordinates and B-factors were refined using 400 cycles of jelly-body refinement in REFMAC5 (Murshudov et al., 2011) as implemented in CCP-EM (Nicholls et al., 2018, Burnley et al., 2017). Convergence of the refinement was assessed using FSC_avg_ (the average model-to-map FSC value). To test the model’s agreement with both the RELION and SIDESPLITTER reconstructions, new post-processed volumes were made without any FSC-weighting or low-pass filtering and using a mask containing only the protein region (i.e. excluding the micelle). The orientation of the two reconstructions had drifted apart during 3D refinement, so in order to make a fair comparison of the model fit, the SIDESPLITTER reconstruction was aligned and resampled onto the same grid as the RELION reconstruction using UCSF Chimera. Model-to-map FSC curves were calculated by REFMAC5 using the “Model validation” task in CCP-EM (Supplementary Fig. 3).

### 2.8 SIDESPLITTER reference implementation details

We provide a reference implementation of SIDESPLITTER as an optimised C99 program using FFTW3 for Fourier transformation (Frigo and Johnson, 2005) to maximise speed and portability. SIDESPLITTER operates upon MRC mode 2 format maps (Cheng et al., 2015), i.e. C float or FORTRAN real. Source code for the SIDESPLITTER reference implementation is available from the Imperial College Section for Structural Biology GitHub (github.com/StructuralBiology-ICLMedicine) under the GPL open source licence. SIDESPLITTER can be compiled for any POSIX-compatible operating system, and will also be made available in pre-compiled binary format for both Linux and Mac OS X as part of the CCP-EM suite (Burnley et al., 2017). A script to run SIDESPLITTER in the context of a RELION 3.1 refinement job is provided alongside the source code.

## 3 Results

### 3.1 Synthetic data demonstrates that regions of low local resolution remain prone to residual over-fitting during single particle refinement of independent half-sets

**Fig. 2:**
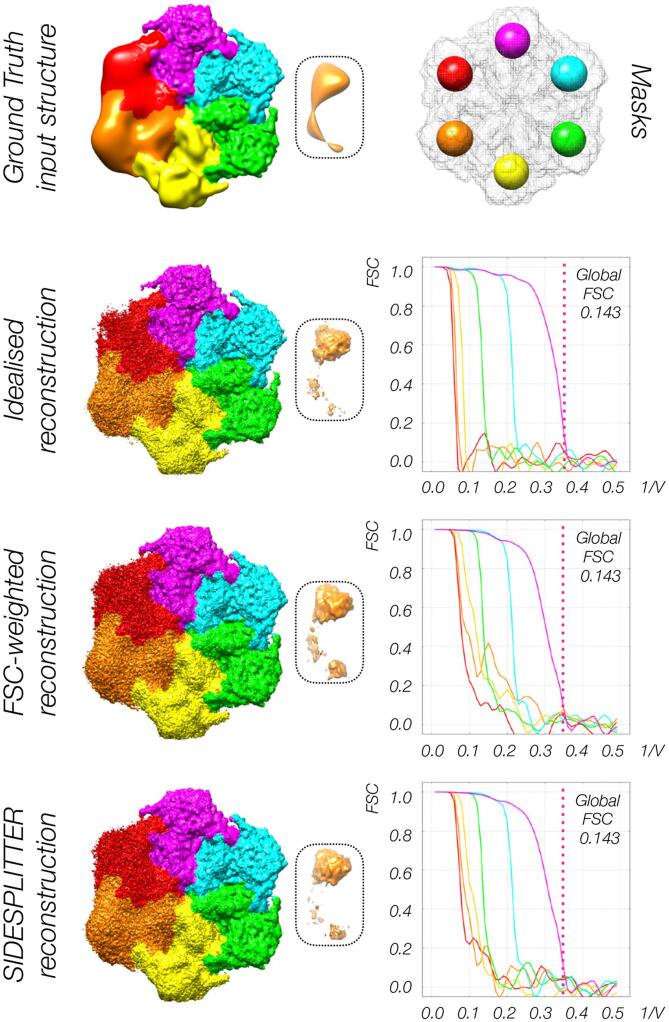
Refinement of synthetic data of known local resolution demonstrates that over-fitting occurs during independent half-set refinement, and that SIDESPLITTER refinement reduces over-fitting. Panels indicate the ground-truth (the known input structure), the output of idealised refinement against ground-truth, the output of standard independent refinement, and the output of refinement with SIDESPLITTER. Panels inset show density peaks in low local resolution. Over-refinement manifests as over-emphasis upon these peak regions. All volumes are shown as surfaces. The FSC curves between half-sets within soft spherical masks isolating part of each segment are inset, coloured according to the rainbow from red to purple (0.0125 to 0.35 voxels per cycle respectively). The corresponding masks are shown above, within the overall mask used during refinement (in grey).

Over-fitting remains an issue within regions that have a lower local resolution than those at the highest resolution in the reconstruction, even during independent half-set refinement. To demonstrate this, we generated synthetic data so that we could explicitly define and control the SNR of the underlying structure (Fig. 2). It is important to note that this does not duplicate the situation in experimental data, which represents a more complex superimposition of different conformations, however it is impractical to generate such a pseudo-realistic dataset with the clearly defined parameters we require for this experiment. We generated density for a molecular model with six-fold symmetry, and truncated the resolution of each subunit to a different resolution between 0.0125 and 0.35 cycles per voxel (Methods section 2.5). Knowing the exact properties of the underlying structure allows us to conclude that any correlation between the datasets beyond the expected resolution is due to noise retained during the refinement process, as there is known to be no initial signal to recover.

On refinement with the standard global filtering approach, while we observed no over-fitting beyond the known global resolution cut-off (0.35 cycles per voxel), there was evidence of overfitting beyond the known resolution of the signal within the segments of low local resolution (Fig. 2, FSC-weighted reconstruction). In particular, the strength of the density of peaks within regions of lower local resolution was over-estimated after standard refinement (Fig. 2. insets). These observations were confirmed by the FSC curves between masked regions in each segment. The FSC curves for the regions of low local resolution fall off from the known point of truncation, but exhibit residual correlation to higher resolutions (Fig. 2). For example, the orange curve remains significantly above zero at 0.3 cycles per voxel, higher than the ground truth (known input structure) resolution of 0.025 cycles per voxel.

### 3.2 Refinement against the input structure yields reconstructions without over-fitting

In order to confirm that the observation of over-fitting is due to the accumulation of noise, we aligned against the ground-truth (known input structure) as the reference volume for each refinement iteration. Identical synthetic data and refinement procedures were used, however the reconstructed half maps were replaced with the synthetic template structure filtered to the current resolution at each iteration. Little-to-no over-fitting was observed beyond the expected cut-off in each segment, both as measured by FSC extension and based on visible features (Fig. 2, idealised reconstruction). Note that all of the FSC curves in this case fall off steeply to near zero, indicating minimal residual correlation at higher resolutions.

### 3.3 Over-fitting is substantially mitigated by application of the SIDESPLITTER algorithm

After confirming that we could reproduce the over-fitting issue under controlled conditions, we attempted to mitigate against it using SIDESPLITTER. When SIDESPLITTER was applied to the half maps between iterations and the corresponding output used for the next iteration of the refinement the FSC curves revealed greater retention of noise at higher resolutions than for the ground-truth case, but substantially less than was the case for the original refinement (Fig. 2, SIDESPLITTER reconstruction). Similarly, excess features were of intermediate strength (Fig. 2, inset), implying that SIDESPLITTER mitigated but did not completely alleviate over-fitting.

### 3.4 Application of the SIDESPLITTER algorithm successfully mitigates against over-fitting in two experimental datasets with low local resolution detergent micelles

Having confirmed the benefit of our approach in principle, we set out to confirm that it was applicable in practice to experimental data. Two experimental conditions were considered: that in which the region of lower local resolution in question is known to lack consistent structure between particles, and that in which there is known and quantifiable heterogeneity between subpopulations of particles. In the first case, we tested SIDESPLITTER using both the recent structure of human amino acid transporter LAT1 bound to CD98 and an antibody fragment within a detergent micelle (EMD-9849; Lee et al., 2019), and with *Pf*CRT bound to an antibody fragment within a detergent micelle (EMD-20806; Kim et al., 2019). Within the micelle the individual detergent molecules are expected to adopt unrelated positions away from the immediate environment of the protein, however some structure often remains apparent within the micelle which is presumably due to over-fitting of noise. Subtraction of the micellar region resulted in a higher-resolution structure of the protein in both the tested cases.

**Fig. 3:**
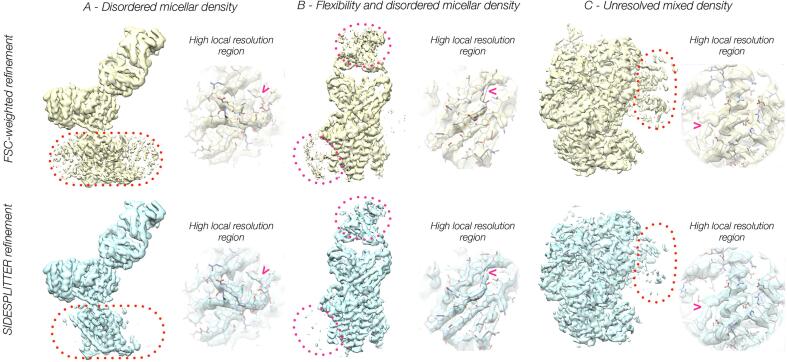
Refinement of experimental datasets with substantial regions of known low local resolution / SNR shows that SIDESPLITTER refinement suppresses features within regions of low local resolution, and improves the quality of the final density. Comparison between the results of standard and SIDESPLITTER refinement for (A) EMD-9849, (B) EMD-20806 and (C) EMD-4038. Volumes are shown as transparent surfaces. Regions of high local resolution are inset in each case, with the corresponding structure fitted into the densities in question, demonstrating that signal is retained to high resolution in regions with a high local signal to noise ratio. Representative improvements in the SIDESPLITTER map are highlighted with arrowheads. In each case the two reconstructions are compared at an identical contour. For the overview images on the left, the contour level was chosen as that sufficient to exclude all background noise, whereas for the inset images on the right the contour level was chosen at the point at which the main chains became clearly interpretable.

In both cases the application of SIDESPLITTER refinement to the original data resulted in a structure with a substantial reduction in both the power of, and the features within, the micelle (Fig. 3A/B, S4), and an improvement in resolution of the protein that was comparable to the micelle-subtraction approach. The quality and connectivity of the density within the better ordered regions of the structure was also notably improved.

### 3.5 Application of the SIDESPLITTER algorithm successfully down-weights regions of an experimental dataset known to correspond to multiple conformational states

In a second experimental case, a RecBCD dataset (EMD-4038; Wilkinson et al., 2016), we ran a single reconstruction combining particles that had previously been split into four classes, in which a rotationally averaged movement of one domain is very evident. We would expect a loss of features within these regions if our approach is successful, as alignment over the rotationally averaged region will reduce the accuracy of the alignment of the core regions. The application of SIDESPLITTER refinement to this data resulted in a reduction of visible features and power within this region exactly as we expected (Fig. 3C), and improved density within the core region as would be predicted given greater angular accuracy.

### 3.6 The SIDESPLITTER algorithm does not degrade the final resolution limit attained, and will yield higher resolution in cases in which over-fitting is severe

For all three experimental applications, and in other tests performed to date (data not shown), density from SIDESPLITTER refinement appears to be clearer and cleaner than that from standard refinement (Fig. 3). For the dataset exhibiting unresolved heterogeneity, the apparent resolution by FSC was identical to that in the case of standard refinement, implying that SIDESPLITTER is not derogatory to the overall resolution attained. For the micellar cases, the resolution according to FSC 0.143 is higher than in the case of standard globally filtered refinement. For LAT1, we obtain 3.2 Å with SIDESPLITTER versus 3.3 Å for RELION FSC-weighted refinement. For PfCRT, we obtain 3.4 Å with SIDESPLITTER versus 3.8 Å for RELION FSC weighted refinement (for reference, Punjani *et al.* reported 6.9 Å for global refinement of this data set with cryoSPARC, and 3.6 Å for non-uniform refinement). Our improved resolutions with SIDESPLITTER are at least equivalent to the improvements seen when subtraction of the micelle has been performed (Lee et al., 2019, Kim et al., 2019). In order to demonstrate that map quality had been improved, and that the effects on resolution and FSC were not simply due to suppression of the weaker regions, we compared the RELION and SIDESPLITTER maps against a molecular model. We refined a model of LAT1 against the RELION FSC-weighted map, and then compared the cross-FSC of the fitted molecular model with the SIDESPLITTER map. The cross-FSC to the SIDESPLITTER reconstruction (against which the model was not refined) was greater than that to the RELION reconstruction (against which the refinement had taken place) within the resolution range of the molecular refinement, conclusively demonstrating that the SIDESPLITTER density was an improved representation of the molecule (Fig. S3).

## 4 Discussion

Over-fitting within macromolecular structures is particularly pernicious, as it undermines the interpretation of biological structure and function. If, within regions of a reconstruction, the noise dominates, it can be mistaken for signal, rendering any interpretation necessarily flawed. The twin problems of the resolution at which a reconstruction remains interpretable, and of variable local “resolution” or SNR, have been investigated heavily. Independent half-set refinement, in which over-fitting is mitigated against during the refinement process (Grigorieff, 2000, Scheres and Chen, 2012, Henderson et al., 2012), local resolution measurement (Cardone et al., 2013, Kucukelbir et al., 2014) and local resolution filtering (Cardone et al., 2013, Vilas et al., 2018a), pursued after the refinement process, have all been widely adopted to avoid over-interpretation of reconstructed densities.

Here we have shown that, despite these advances, over-fitting during the refinement process within areas of low local resolution / SNR remains problematic during independent half-set refinement. The two half sets contributing to the reconstructions can be kept independent; however, the noise within the resulting reconstructions will not be uncorrelated. Despite the separation of the two sets of particle images, certain characteristics are shared between the sets, including the regions of lower local resolution (and corresponding local high-resolution noise), the orientation distribution, initial model, and the mask used in refinement. This means that density corresponding to noise will tend to accumulate similarly, even if independently, on each side of a split refinement. This process leads inexorably to over-fitting in more poorly-resolved regions of the reconstructed density through the positive feedback process of iterative refinement, noise being aligned against noise. Such over-fitting cannot be entirely mitigated against after the refinement, as the incorporated noise becomes indistinguishable from signal. Affected regions will have higher apparent SNR, and exhibit higher apparent resolution, than should be the case given the underlying data, and flawed interpretation of such structures is a real risk.

Remedies for this using some form of local filtering have been proposed previously, and basic implementations provided (Grigorieff, 2016) for simple versions of weighting approaches with user intervention. In looking for an algorithm to allow automatic and unbiased weighting along these lines, the major (and non-trivial) problem is to maintain the independent nature of the split refinement. The application of windowed local-resolution filters cannot maintain this independence, as the shared signal within local windows must necessarily become correlated, and therefore such filters cannot be compatible with an independent split refinement (Supp. Fig. 1). We have overcome this issue by creating a local SNR filter suitable for independent refinement of a split dataset. SIDESPLITTER, based on a modified local SNR filter that minimises the residual noise within the two reconstructions (Ramlaul et al., 2019), maintains the independence of the two sides of a split refinement by taking account of only the global noise distribution between them.

During the publication process for this manuscript, Punjani and colleagues (Punjani et al., 2019 – preprint) have proposed an alternative local filtering approach for use in refinement. Under their approach separate local resolution weights are generated between two quarter datasets for each half of a refinement split into four separate datasets, allowing independence between two halves of the refinement to be maintained for the purposes of FSC calculations. We agree that this is also a viable approach to the problem, however we note that their approach requires double the number of single particle refinements in comparison to SIDESPLITTER, and that each of the two sets of local resolution weights will be generated with only half the statistical power that is available to SIDESPLITTER.

We have shown that SIDESPLITTER effectively mitigates over-fitting both in synthetic situations, where we have explicitly generated and measured over-fitting, and in experimental data with known over-fitting problems which have previously been mitigated by the manual interventions of particle sorting and density subtraction. One natural consequence of this approach is that regions of disagreement will exhibit weaker density in the resulting reconstruction. Our approach is motivated by the principle that the alignment to the largest rigid body within the reconstruction should be paramount. Misalignment to any flexible subdomain will necessarily either introduce artefacts or reduce the final resolution, and often both. We consider such artefacts within the rigid region undesirable, even in exchange for a slightly higher resolution or SNR representation of the flexible domain, especially when such flexible regions are typically disrupted and difficult to interpret under the best of circumstances.

The statistical approach used in RELION, through which we have tested SIDEPSLITTER refinement, requires an estimate of the signal-to-noise ratio (SNR) in the current reference map. When the “use solvent-flattened FSC” option is enabled, this is calculated from the FSC of the masked half maps (Scheres, 2012b) with a correction for the mask-induced correlation by phase randomization (Chen et al., 2013). This ignores the improvement by SIDESPLITTER and underestimates the SNR in the new reference, leading to underestimation of the angular accuracy. In one of our examples, the *Pf*CRT dataset, this led to premature termination of the auto-refinement procedure, necessitating user intervention. Better integration of SIDESPLITTER with RELION and other refinement programs remains a topic of ongoing research.

SIDESPLITTER refinement has shown demonstrable improvements in the output density in situations where there are large regions of lower local resolution, and the reduction of over-fitting has been shown to increase the overall resolution in particularly egregious cases, where regions of low local resolution make up a substantial portion of the refined density. The improved angular accuracy available after SIDESPLITTER refinement will also be highly beneficial for downstream processing steps including classification without alignment and density subtraction. We believe that the SIDESPLITTER approach will be of benefit to the field during any refinement in which there is a notable variation in local resolution within the volume.

## CRediT authorship contribution statement

**Kailash Ramlaul:** Methodology, Validation, Data curation, Writing - original draft, Writing - review & editing, Visualization. **Colin M. Palmer:** Methodology, Software, Validation, Investigation, Resources, Data curation, Writing - review & editing, Visualization. **Takanori Nakane:** Methodology, Software, Validation, Investigation, Resources, Data curation, Writing - review & editing. **Christopher H.S. Aylett:** Conceptualization, Methodology, Software, Validation, Formal analysis, Investigation, Resources, Data curation, Writing - original draft, Writing - review & editing, Visualization, Supervision, Project administration, Funding acquisition.

## Declaration of Competing Interest

The authors declare that they have no known competing financial interests or personal relationships that could have appeared to influence the work reported in this paper.
