# Supplementary material for: Mitigating local over-fitting during single particle reconstruction with SIDESPLITTER
Source: J Struct Biol. 2020 Aug 1;211(2):107545. doi: 10.1016/j.jsb.2020.107545 (PMC7369633; doi:10.1016/j.jsb.2020.107545)
Supplement: Supplementary data 1 [file mmc1.pdf]

Mitigating Local Over-fitting During Single Particle Reconstruction with SIDESPLITTER

Kailash Ramlaul[1*], Colin M. Palmer[2*], Takanori Nakane[3] & Christopher H. S. Aylett[1†]

[1] Section for Structural and Synthetic Biology, Department of Infectious Disease, Faculty of Medicine, Imperial College, Imperial College Road, South Kensington, London, SW7 2BB, United Kingdom

[2] Scientific Computing Department, Science and Technology Facilities Council, Research Complex at Harwell, Didcot, OX11 0FA, United Kingdom

[3] Medical Research Council Laboratory of Molecular Biology, Cambridge CB2 0QH, United Kingdom

* These authors contributed equally to this work

† To whom correspondence may be addressed: c.aylett@imperial.ac.uk

Supplementary Figures and Figure Legends

S1

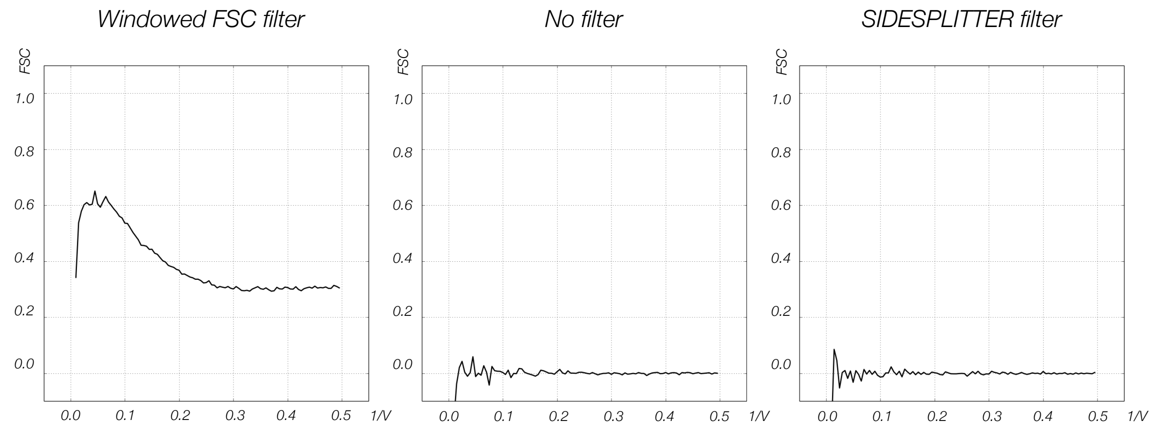

Windowed local filters and shared local resolution maps generate spurious correlations between uncorrelated white noise half maps, making them unsuitable for independent split refinement

Supplementary Figure 1: Windowed local filters and shared local resolution maps are unsuitable for independent split refinement as they generate spurious correlations. Half-set FSC curves after the application of a windowed FSC filter (BLOCRES/BLOCFILT) (left), without any processing (centre), and after the application of SIDESPLITTER (right) to the same uncorrelated white noise densities. The curves oscillate around zero where a correlation has not been generated by the local filtering process. The BLOCRES/BLOCFILT filter introduces correlation over the entire resolution range. By contrast, the SIDESPLITTER filter introduces no discernible correlation.

S2

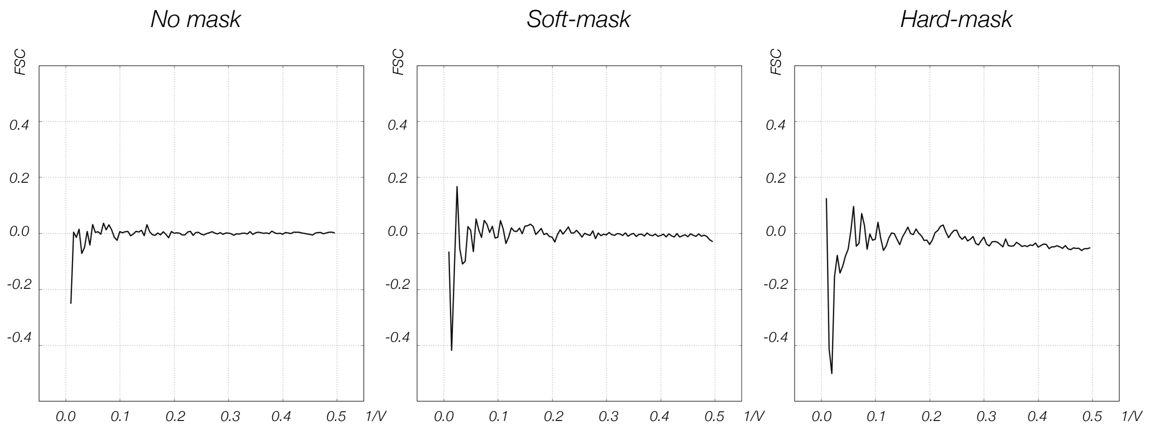

Masking with an identical mask generates correlations between uncorrelated white noise half maps, which can be minimised by the use of a less-featured, "softer" mask

Supplementary Figure 2: Shared solvent masks also generate spurious correlations, although the effect is not pronounced, especially for soft masks. Resulting half-set FSC curves after the application of no mask (left), a soft mask (centre), and a relatively sharp mask (right) to the same uncorrelated noise densities. The curves oscillate around zero where a correlation has not been generated by the local filtering process. In the unmasked noise half-maps, oscillations indicating correlations are much smaller in magnitude compared to the masked half-maps. The hard-masked maps in particular display aggravated correlations at low resolution and some correlation at high resolution. The correlations in the soft-masked maps are smaller and occur only at low resolution, but they are still noticeable, indicating that artefactual correlations due to masking can be minimised, but not abrogated, by using softer masks.

S3

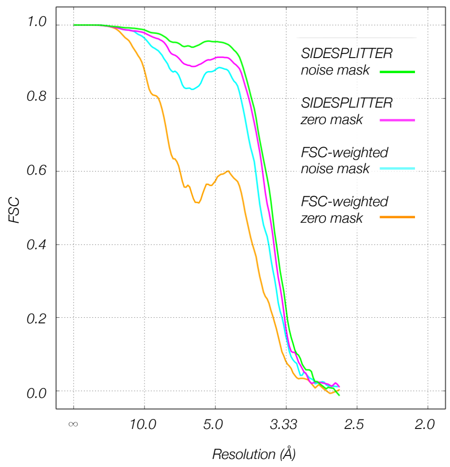

Both noise masking as opposed to zero masking and SIDESPLITTER refinement substantially improve both FSC and resolution extension at convergence

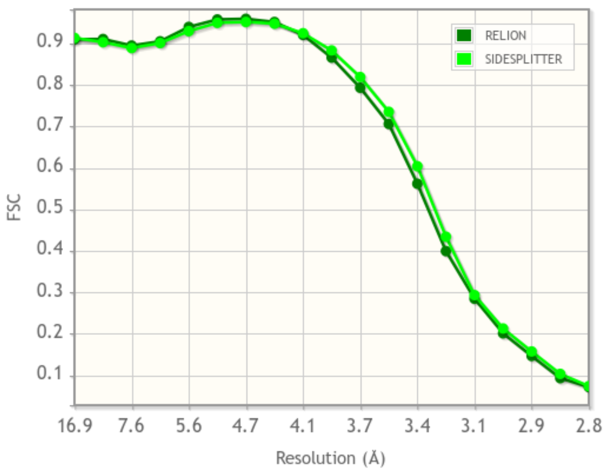

SIDESPLITTER refinement improves map quality - a model refined into the FSC-weighted RELION refinement exhibited a greater cross-FSC with the SIDESPLITTER reconstruction than its cognate map

Supplementary Figure 3: SIDESPLITTER refinement improves the map and molecular model fit for LAT1. Left panel: Both masking with the noise distribution instead of zeros, and SIDESPLITTER refinement substantially improve the FSC between independent half sets, and these effects are cumulative. *Right panel:* the cross-FSC of a model refined against the FSC weighted RELION reconstruction to the SIDESPLITTER reconstruction (against which the model was not refined) was greater than that to the RELION reconstruction (against which the model had been refined) within the resolution range against which the coordinate refinement had taken place.

S4

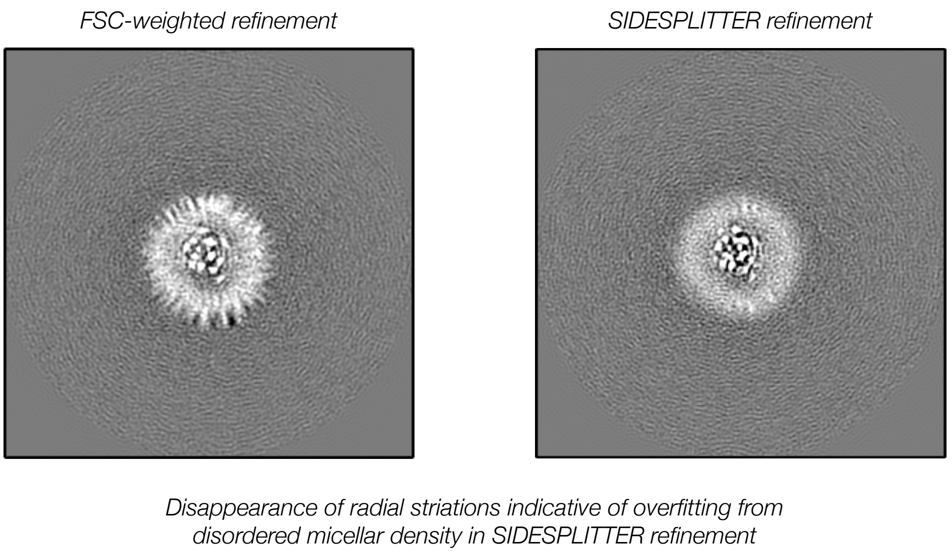

Supplementary Figure 4: SIDESPLITTER refinement results in the suppression of striations due to overfitting. Sections through the micellar density showing visible evidence of overfitting in standard refinement, which are suppressed on SIDESPLITTER refinement.
